# Humanized-V_H_H Transbodies that Inhibit HCV Protease and Replication

**DOI:** 10.3390/v7042030

**Published:** 2015-04-20

**Authors:** Surasak Jittavisutthikul, Jeeraphong Thanongsaksrikul, Kanyarat Thueng-in, Monrat Chulanetra, Potjanee Srimanote, Watee Seesuay, Aijaz Ahmad Malik, Wanpen Chaicumpa

**Affiliations:** 1 Graduate Program in Immunology, Department of Immunology, Faculty of Medicine Siriraj Hospital, Mahidol University, Bangkok 10700, Thailand; E-Mails: toeyku61@hotmail.com (S.J.); ajaz_me@hotmail.com (A.A.M.); 2 Laboratory for Research and Technology Development, Department of Parasitology and Center of Excellence on Therapeutic Proteins and Antibody Engineering, Faculty of Medicine Siriraj Hospital, Mahidol University, Bangkok 10700, Thailand; E-Mails: gskmu@hotmail.com (J.T.); mam_mt41@hotmail.com (K.T.); monchu82@gmail.com (M.C.); watee.see@gmail.com (W.S.); 3 Graduate Program in Biomedical Science, Faculty of Allied Health Sciences, Thammasat University, Pathum-thani 12120, Thailand; E-Mail: srimanote_p@y7mail.com; 4 Department of Microbiology and Immunology, Faculty of Veterinary Medicine, Kasetsart University, Bangkok 10900, Thailand

**Keywords:** Anti-hepatitis C virus (HCV) agent, HCV protease, homology modeling and molecular docking, nanobody, V_H_H, phage display, transbody

## Abstract

There is a need for safe and broadly effective anti-HCV agents that can cope with genetic multiplicity and mutations of the virus. In this study, humanized-camel V_H_Hs to genotype 3a HCV serine protease were produced and were linked molecularly to a cell penetrating peptide, penetratin (PEN). Human hepatic (Huh7) cells transfected with the JFH-1 RNA of HCV genotype 2a and treated with the cell penetrable nanobodies (transbodies) had a marked reduction of the HCV RNA intracellularly and in their culture fluids, less HCV foci inside the cells and less amounts of HCV core antigen in culture supernatants compared with the infected cells cultured in the medium alone. The PEN-V_H_H-treated-transfected cells also had up-regulation of the genes coding for the host innate immune response (*TRIF*, *TRAF3*, *IRF3*, *IL-28B* and *IFN-β*), indicating that the cell penetrable nanobodies rescued the host innate immune response from the HCV mediated-suppression. Computerized intermolecular docking revealed that the V_H_Hs bound to residues of the protease catalytic triad, oxyanion loop and/or the NS3 N-terminal portion important for non-covalent binding of the NS4A protease cofactor protein. The so-produced transbodies have high potential for testing further as a candidate for safe, broadly effective and virus mutation tolerable anti-HCV agents.

## 1. Introduction

Hepatitis C virus (HCV) infection is usually manifested as a chronic liver inflammation which, over time, progresses to fatal cirrhosis and hepatocellular carcinoma. At present, there is no effective vaccine against the infection. A standard-of-care (SOC) for the infected patients is weekly injected pegylated-interferon alfa (PEG-IFN-α) in combination with daily oral ribavirin (a nucleoside analog) for rescuing the host immunity, mitigating the clinical symptoms and controlling the viral load [1]. However, this dual therapy is not only prolonged and stringent but also causes severe adverse effects including flu-like symptoms (fever and fatigue), neuropsychiatric disorder, hematologic complications and/or autoimmune condition and thus received low patients’ compliance [2]. Moreover, strains of genotypes 1 and 4 are relatively refractory to the SOC [3]. Thus, there is a need of a more effective and side effect-free anti-HCV agents. During the past decade, intense efforts have been made for developing novel small molecules that target HCV proteins, so-called direct acting antiviral agents (DAAs). Examples are inhibitors of the HCV protease (e.g., boceprevir, telaprevir, simeprevir, paritaprevir, asunaprevia and faldaprevir), polymerase (e.g., sofosbuvir and dasabuvir), NS4B (e.g., substituted imidazo(1,2-α)pyrimidines) and NS5A (e.g., ombitasvir, daclatasvir) [4,5,6,7]. Triple therapy (one protease/polymerase inhibitor + SOC) conferred higher percentage of sustained virologic response (SVR) compared with the SOC alone. Nevertheless, they encounter several limitations including additional severe adverse effects as well as drug-drug interaction [8,9]. They are contraindicated for patients with underlying conditions such as decompensated liver/metabolic diseases, autoimmune conditions, chronic diseases of kidney, heart and lungs and pregnancy [2]. Besides, the protease/polymerase inhibitors cannot be used alone as it causes emergence of DAA-resistant mutants which also exhibit cross-resistance to multiple drugs [7]. Thus, monotherapy with the HCV inhibitors should be absolutely avoided [7]. More recently, interferon-free cocktails of oral DAAs (similar strategy to the precedent combined antiviral regimens for treatment of HIV infections) were found to be highly effective for treatment of HCV especially for patients with genotype 1b infection and limited treatment options [10,11].

HCV has a positive-sense, single stranded-RNA genome that encodes a polyprotein of about 3000 amino acids. The newly produced polyprotein is co- and post-translationally processed by the host and viral proteases into four structural and six non-structural (NS) active proteins, *i.e*., core, E1, E2 and p7 (viroporin) and NS2, NS3, NS4A, NS4B, NS5A and NS5B, respectively [12,13]. The HCV NS3 is a multifunctional protein. The N-terminal, one-third (180 amino acids), of the molecule in combination with the NS4A (NS3/4A) is an active trypsin/chymotrypsin-like serine protease which catalyzes cis-cleavage at the NS3-NS4A junction and trans-cleavage at all of the down-stream junctions into mature NS4A, NS4B, NS5A and NS5B [14]. The protease activity requires a catalytic triad (H57, D81 and S139), an oxyanion loop (L135, K136, G137, S138 and S139) and the zinc binding domain (C97, C99, C145 and H149) of the HCV NS3 [15]. The consensus cleavage sequence of the HCV protease is D/EXXXXC/T↓S/AXXX. The enzyme also cleaves the key host adapter proteins, *i.e*., MAVS (IPS-1/CARDIF/VISA) and TRIF resulting in ablation of the RIG-I- and TLR-3-mediated innate immune signaling [16,17].

Each antibody molecule binds to several residues/spatially juxtaposed portions (conformational epitope) of the target antigen by using multiple complementarity determining regions (CDRs) as well as canonical immunoglobulin framework regions (FRs) [18]. Contemporary technology has made possible an *in vitro* production of engineered antibodies in a variety of formats including intact IgG and antibody fragments, *i.e*., Fab, single chain antibody (ScFv) and single domain antibody (sdAb; VH/V_H_H or nanobody) [19,20,21]. The antibody for human therapeutic use can be made into fully human or humanized molecules with negligible immunogenicity in the human recipients; thus, they are safe (no induction of anti-isotype response and the adverse sequels) [22]. A cocktail of monoclonal antibodies specific to multiple epitopes/proteins of the pathogen can be prepared *in vitro* without a prolonged immunization process and *in vivo* immune regulations and they can be readily standardized. In this study, cell penetrable humanized-camel V_H_Hs (transbodies) that bound specifically to the HCV protease were produced. Ability of the transbodies to interfere with the heterologous HCV replication was studied. It is envisaged that a right mixture of human/humanized-cell penetrable small antibodies specific to different epitopes of HCV highly conserved vital enzymes/proteins should be a safe, broadly effective, relatively mutation tolerable anti-HCV agent.

## 2. Materials and Methods

### 2.1. Production of Recombinant HCV NS3 and NS4A Fusion Protein (rNS3/4A)

The recombinant NS3/4A fusion protein containing N-terminal 180 amino acids of the NS3 and residues 21–32 of the NS4A protein was produced. HCV genomic RNA was extracted from serum samples of patients infected with genotype 3a (predominant genotype in Thailand). The RNA was reverse transcribed to cDNA using oligo(dT) primer and the preparation was used as a template for amplification of the *NS3/4A* sequence by spliced overlapped extension-polymerase chain reaction (SOE-PCR) using oligonucleotide primers specific to NS3/4A coding regions designed from NS3 and NS4A nucleotide sequences of HCV genotype 3a (accession no. NC_009824). The primers were: F-1:5'-CAT ATT GAG CTG GAG GGT AGT GGT AGT GGC CGT GAG GTG TTG TTG-3', F-2:5'-GGA TCC TGG CTG CGT TGT GAT TGT GGG TCA TAT TGA GCT GGA G-3' and R: 5'-CTC GAG ATA GCT CTG TGG AAC AGC AGG AGG-3'. *Bam*HI and *Xho*I restriction sites (underlined) were included in the primer sequences for facilitating subsequent gene cloning. The DNA amplicon was cloned into pET23b^+^ vector between the *Bam*HI and *Xho*I sites and the recombinant plasmid was introduced into BL21 (DE3) *E. coli.* A transformed *E. coli* colony on the selective agar plate was picked and grown under 0.3 mM isopropyl-β-D-1-thiogalctopyranoside (IPTG) induction condition and the expressed recombinant protein (rNS3/4A) with 6× His tag at the C-terminal was purified under denaturing condition from the *E. coli* inclusion by using TALON® metal affinity resins (Clontech, Mountain View, CA, USA) and verified by LC-MS/MS.

### 2.2. Determination of Protease Activity of the rNS3/4A

Serine protease activity of the rNS3/4A was determined by using SensoLyte® 490 HCV Protease Assay Kit (AnaSpec, Freemont, CA, USA). The rNS3/4A ability to cleave an engineered substrate was monitored by a continuous fluorescence resonance energy transfer (FRET) method [23]. The fluoresecent peptide substrate of the NS3/4A protease used in the reaction contained nine amino acids covering the HCV NS3 protease cleavage site on the polyprotein except the cysteine residue was replaced with aminobutyric acid, *i.e*., DE-D(Edans)-EE-Abu-ψ-[COO]-AS-K-(Dabcyl). The EDANS fluorescence donor was quenched by DABCYL in the intact peptide. In the presence of active protease, the peptide was cleaved at the Abu-ψ-Ala junction into two separate fragments DE-D(Edans)-EE-Abu-ψ-COOH and AS-K- DABCYL. The released EDANS fluorescence could be monitored at the excitation/emission = 340/490 nm by spectrofluorometer. Triplicate wells of a 96-well black plate (Costar, Corning, Tewksbury, MA, USA) were added individually with 5 μg of rNS3/4A (from titration) and incubated at 37 °C for 15 min. The fluorescence substrate was added to the wells and the plate was immediately placed in a VarioSkan Flash®microplate reader (Thermo Fisher Scientific, Waltham, MA, USA) for measuring the enzymatic reaction (fluorescence intensity) during 30 min at 2 s intervals. Triplicate wells containing buffer and substrate served as negative (background) control. The average background fluorescence intensity was used to subtract the average value of the test to get the relative fluorescence units (RFU). A progressive curve was drawn for reaction at different time points. The initial reaction velocity (*v_i_*) in RFU/min was calculated from the reaction mixture by linear regression method. The *v_i_* was fitted with Michaelis-Menten equation by using GraphPad Prism 5 software to calculate maximum velocity (*V_max_*) and *K_m_*. The rNS3/4A with the protease activity was used subsequently as the antigen in a phage bio-panning.

### 2.3. Production and Characterization of Soluble Nanobodies (VHs/V_H_Hs) that Bound to rNS3/4A

A humanized-camel VH/V_H_H phage display library used in this study was constructed previously [21]. Total RNA was extracted from lymphocytes of an eight month old naїve camel, *Camelus dromedarius*, and reverse transcribed to cDNA. The cDNA was used as template for amplification of the camel conventional *vh* and heavy chain antibody *v_h_h* sequences by PCR using human degenerate primers designed from all families/subfamilies of human immunoglobulin genes [20]. The human primer directed-*vh/v_h_h* amplified sequences (humanized-*vhs/v_h_hs*) were ligated to pCANTAB5E phagemid vector and the recombinant phagemids were used to infect competent TG1 *E. coli* bacteria. After co-infecting the *vh/v_h_h-*phagemid-transfected *E. coli* with a helper phage (M13KO7), the complete phage particles displaying humanized-VHs/V_H_Hs on the surface as fusion proteins with the phage p3 protein and carrying integrated *vhs/v_h_hs* in their genomes (humanized-camel VH/V_H_H phage display library) were obtained from the bacterial culture supernatant.

Phage bio-panning for selecting phage clones that bound to rNS3/4A was performed as described previously [24,25]. Ten μg of the purified rNS3/4A was coated onto an ELISA well surface and the humanized-camel VH/V_H_H phage display library was added to the well. After an incubation, antigen unbound phages were removed by washing thoroughly and the bound phages were immediately supplemented with a log phase grown HB2151 *E. coli*. The phagemid transformed *E. coli* clones were grown overnight on a selective agar plate (Luria-Bertani agar containing 100 μg/mL amplicillin and 2% glucose; LB-AG). Phagemid transformed *E. coli* clones were randomly selected and screened for the presence of recombinant *vh/v_h_h*-phagemids by PCR using the *R1* and *R2* primers [20]. The *E. coli* carrying *vh/v_h_h*-phagemids were grown individually under 0.5 mM IPTG induction for production of soluble E-tagged-VHs/V_H_Hs. The presence of the nanobodies in the bacterial lysates was detected by SDS-PAGE and Western blot analysis using rabbit anti-E Tag antibody (Abcam®, Cambridge, UK) as a detection reagent. Goat anti-rabbit immunoglobulin-alkaline phosphatase (AP) conjugate (Southern Biotech, Birmingham, AI, USA) was used as an anti-isotype antibody and BCIP/NBT^TM^ substrate (KPL) was used as a chromogenic substrate. The humanized-VHs/V_H_Hs in the *E. coli* lysates were purified by using DEAE-Sepharose^TM^ Fast Flow packed beads (Pharmacia, Stockholm, Sweden). The amounts of the VHs/V_H_Hs in the *E. coli* lysates were standardized before testing for binding to the homologous antigen by indirect ELISA and Western blot analysis.

For indirect ELISA, one μg aliquots of the purified rNS3/4A were immobilized in wells of an ELISA plate. Wells coated with 1% bovine serum albumin (BSA) (control antigen) in PBS and coating buffer only (blank) were included in the assay. After blocking the empty sites on the well surface with 3% BSA in PBS, individual *E. coli* lysates containing standardized VHs/V_H_Hs and lysate of original HB2151 *E. coli* (negative antibody control) were added to appropriate wells and incubated at 37 °C for 1 h. Unbound materials were removed; rabbit anti-E Tag (Abcam®), goat anti-rabbit immunoglobulin-horseradish peroxidase (HRP) conjugate (Southern Biotech) and ABTS substrate (KPL) were added sequentially with washing between the steps. VHs/V_H_Hs in *E. coli* clones that revealed optical absorbance at 405 nm (OD_405_) to rNS3/4A at least two times higher than to BSA control were selected.

For Western blot analysis, purified rNS3/4A (10 μg) was subjected to 12% SDS-PAGE and the separated components were blotted onto a nitrocellulose membrane (NC). The blotted NC was cut vertically into strips and blocked with 5% skim milk in Tris buffered saline (TBS) containing 0.05% Tween-20 (TBST). The strips were probed individually with *E. coli* lysates containing the VHs/V_H_Hs at 25 °C for 1 h. A lysate of original HB2151 *E. coli* was used as negative VH/V_H_H control. After washing, the antigen-antibody reactive band on each NC strip was detected by using rabbit anti-E Tag, goat anti-rabbit immunoglobulin-AP conjugate and BCIP/NBT^TM^ substrate, respectively. The rNS3/4A blotted strip probed with the antibody diluent incubated with mouse anti- 6×His Tag, goat anti-mouse immunoglobulin-AP conjugate and BCIP/NBT^TM^ substrate served as positive reaction control.

Restriction fragment length polymorphism of *vh/v_h_h* sequences from the selected HB2151 *E. coli* clones were determined [25]. Briefly, antibody coding sequences were PCR amplified by using the *R1* and *R2* primers and digested with *Mva*I restriction endonuclease. The digested mixtures were resolved individually in 14% polyacrylamide gel containing 0.5% glycerol followed by ethidium bromide staining. The DNA banding patterns were visualized under UV transilluminator (Bio Doc-IT^TM^ Imaging System, UVP, Upland, CA, USA). The *vh/v_h_h* sequences of the phagemid-transformed HB2151 *E. coli* clones were also subjected to DNA sequencing. Deduced amino acid sequences of all clones were multiply aligned by ClustalW. The CDRs and FRs of individual VHs/V_H_Hs were predicted by using the International ImMunoGeneTics (IMGT®, Montpellier, France) information system.

### 2.4. Production of Cell-Penetrable Nanobodies (Transbodies)

The *vh/v_h_h* sequences of *E. coli* clones of interest were subcloned from the pCANTAB5E phagemid vector to pET23b^+^ backbone carrying a DNA insert coding for a 16 amino acid cell penetrating peptide (CPP), named penetratin (PEN) [26] for making cell penetrable nanobodies (transbodies). The recombinant *pen-vh/v_h_h*-pET23b^+^ plasmids were introduced into *E. coli* BL21 (DE3). PEN-VH/V_H_H fusion proteins were produced from selected transformed *E. coli* clones grown under 0.5 mM IPTG induction for 3 h. The recombinant PEN-VHs/V_H_Hs with 6× His tag were purified from the *E. coli* homogenates by using TALON® metal affinity resins (Clontech, Inc.). They were tested for cell penetrating ability and toxicity to human hepatic (Huh7) cells.

### 2.5. Cell Penetrating Ability of the PEN-VHs/V_H_Hs

Cell penetrating ability of the PEN-nanobodies was determined by indirect ELISA and confocal microscopy as described previously [26,27]. The Huh7 cells were cultured in DMEM supplemented with 10% heat inactivated fetal calf serum (Hyclone, South Logan, TX, USA), penicillin (50 units/mL) and streptomycin (50 μg/mL). The grown cells were added to cover slips placed in wells of a 96 well tissue culture plate (2 × 10^4^ cells/slip) and incubated at 5% CO_2_ incubator at 37 °C for 24 h. The Huh7 monolayer were washed with sterile PBS and then incubated with 10 µg of individual PEN-nanobody preparations for 1 h. After discarding the culture supernatants, the cells were rinsed with plain DMEM, added with a fixed volume of PBS, homogenized and the lysates were collected after centrifugation. The amounts of PEN-nanobodies in the cell lysates were quantified by indirect ELISA as described previously [25]. For the confocal microscopy, the Huh7 cells that had been incubated with 10 µg of PEN-nanobodies were washed with PBS before fixing with cold methanol for 20 min followed by washing again and then permeated with 1% Triton X-100 for 30 min. The cells were blocked with 3% BSA, washed and incubated sequentially with mouse anti-6× His tag (1:1000) and donkey anti-mouse immunoglobulin (DyLight® 488) (1:1000) with washing between the steps. DAPI (Invitrogen, Calsbad, CA, USA) was used to locate the cell nuclei. The stained cells were observed under a confocal microscope.

### 2.6. LDH Assay

CytoTox96® non-radioactive cytotoxicity (lactate dehydrogenase, LDH) assay kit (Promega, Madison, WI, USA) was used for determining toxicity of the HCV protease specific-transbodies [25,26,27]. The Huh7 cells were cultured as above; 2 × 10^4^ cells were added to individual wells of a 96 well tissue culture plate and incubated at 5% CO_2_ incubator at 37 °C for 24 h. The monolayer were washed twice with PBS before incubating with 25 µg PEN-V_H_Hs for 24 h. Cells incubated with medium alone and 10% SDS were included as negative and positive cytotoxic controls, respectively. The culture supernatant of each well (triplicate wells of each treatment) was collected. The LDH amounts in the fluids were determined and the percent cytotoxicity was calculated: (LDH release of test – LDH release of negative control)/(LDH release of positive control – LDH release of negative control) × 100.

### 2.7. Inhibition of HCV Replication by HCV Protease Specific Cell Penetrable Nanobodies

Genomic HCV RNA was produced from the pJFH-1 containing full-length cDNA of JFH-1 HCV genotype 2a (GenBank AB047639) as described previously [25,27]. The pJFH-1 was linearized with *Xba*I and one μg was subjected to transcription *in vitro* by using MEGAscript® T7 transcription kit (Ambion, Calsbad, CA, USA). The JFH-1 RNA (10 μg) was electroporated into 4 × 10^6^ Huh7 cells in 0.4 mL reduced serum medium (Opti-MEM) (Invitrogen) by using a single pulse at 0.27 kV and 100 milli-s. The electroporated cells were immediately added with complete culture medium (DMEM containing 10% heat inactivated-fetal bovine serum (Hyclone)), distributed into wells of a 12-well tissue culture plate (2 × 10^5^ cells per well) and the plate was incubated at 37 °C in 5% CO_2_ atmosphere for 3 h. The cells were rinsed gently with sterile PBS and replenished with complete DMEM containing 25 μg of purified PEN-VHs/V_H_Hs of individual *E. coli* clones. Controls included cells added with either medium alone (negative inhibition control), 25 μg of control (irrelevant) PEN-V_H_H (background inhibition control), 100 units of PEG-IFN-α + 50 nM ribavirin and 0.175 μM telaprevir (VX-950; Selleckem, Houston, TX, USA) (positive inhibition controls). The plates were incubated further for 5 days. Total RNA was extracted from the culture supernatants and the transfected cells by using Trizol^TM^ reagent (Invitrogen). Copy number of HCV 5' UTR (230 bp) in each sample was determined by quantitative real-time RT-PCR [25,27].

### 2.8. Quantitative Real-Time RT-PCR (qRT-PCR)

The amounts of HCV RNA (copy numbers) in the culture supernatants and inside the cells that received different treatments were determined by qRT-PCR using 1-step Brilliant II SYBR green qRT-PCR mastermix (Agilent Technologies, Santa Clara, CA, USA) [27]. A standard curve was constructed from C*_t_* of ten-fold serial dilutions of the pJFH-1 carrying full-length cDNA of JFH-1 HCV genotype 2a (ranged from 2.77 × 10^7^ to 0.02 copies). C*_t_* from each sample was expressed as log_10_ of RNA copies/mL calculated from the standard curve. Data are reported as the average of three independent experiments.

### 2.9. Foci Assay

The amounts of HCV core antigen (C) in the cell culture supernatants and the HCV inside the HCV RNA transfected cells were determined by a foci assay [27]. Briefly, spent culture fluids of the JFH-1 RNA transfected cells that had been treated with the PEN-nanobodies, control PEN-nanobody, PEG-IFN-α+ ribavirin, telaprevir and medium alone for 5 days were collected. The infected cells in each well were rinsed with sterile PBS, fixed with absolute methanol and blocked with 10% BSA before incubating with mouse immune serum to HCV core protein in order to locate the infected cells. After rinsing, the cells were added with goat anti-mouse immunoglobulin-AP conjugate and BCIP/NBT substrate, respectively, with washing between the steps. Untransfected cells were included in the experiments. The HCV foci were observed and counted by using inverted fluorescence microscope, NIS-Element D version 4.10.0.8310 W/camera (Ti-S Intensilight Ri1 NIS-D, Nikon, Japan) at 10× original magnification.

### 2.10. ELISA for Quantification of HCV Core Antigen

Amounts of HCV core protein in culture supernatants of HCV transfected Huh7 cells that received various treatments were determined by using QuickTiter^TM^ HCV Core Antigen ELISA kit (Cell Biolabs, San Diego, CA, USA). Briefly, 225 µL of each sample were added with 25 µL of Triton X-100 solution and incubated at 37 °C for 30 min. A 100 µL aliquot of each inactivated sample and HCV core antigen standards (provided with the test kit) were added to the anti-HCV core antigen-coated plate and incubated at 37 °C for 2 h. After washing all wells were added individually with FITC-conjugated monoclonal anti-HCV core antigen followed by HRP conjugated-anti-FITC monoclonal antibody and HRP substrate. The absorbance at 450 nm of each well was measured by spectrophotometer. The amount of HCV core antigen in a test sample was calculated from a standard curve constructed from the OD_450nm_ of the HCV core standards.

### 2.11. Response of the HCV RNA Transfected Cells to Treatment with Protease Specific-Cell Penetrable Nanobodies

Expressions of various genes of the RIG-1 and TLR3 signaling pathways were determined as described previously [27]. The JFH-1 RNA transfected Huh7 cells were cultured in complete DMEM in a 12-well culture plate (2.0 × 10^5^ cells/well) for 3 h before incubating with 25 µg of individual PEN-nanobodies (transbodies). The experimental controls were transfected cells treated with control PEN-nanobody (background control), 100 units of PEG-IFN-α + 50 nM ribavirin and 0.175 μM telaprevir (positive inhibition controls) and medium alone (transfection control). Five days post-transfection, total RNA was extracted from the cells of all treatments using Trizol® reagent (Invitrogen) and quantified with a Nano-Drop ND-1000 Spectrophotometer (Thermo Fisher Scientific, Waltham, MA, USA). Expression levels of mRNAs of the innate immune response genes including *IRF3*, *TRIF*, *TRAF3*, *IL-28B* and *IFN-β* were determined. Primers specific to *IRF3*, *TRIF*, and *TRAF3* were: *IRF3*: forward 5'-CTTGGAAGCACGGCCTAC-3' and reverse 5'-CGGAAATTCCTCTTCCAGGT-3; *TRIF*: forward 5'-CTTGGAAGCACGGCCTAC-3' and reverse 5'-CGGAAATTCCTCTTCCAGGT-3'; *TRAF3*: forward 5'-CTCACAAGTGCAGCGTCCAG-3' and reverse 5'-GCTCCACTCCTTCAGCAGGTT-3' [27,28]. Primers for *IL-28B*: forward 5'-AAGGACTGCAAGTGCCGCT-3' and reverse 5'-GCTGGTCCAAGACATCCC-3' and *IFN-β*: 5'-GTCTCATTCCAGCCAGTGCT-3' and reverse 5'-TGGCAATTGAATGGGAGGCT-3', were designed from the GenBank (accession nos. Z56281, NM_182919.3, NM_145725, AY129149 and NM_002176, respectively), using Primer3 software [29]. The PCR reaction mixture (12.5 µL) consisted of 1× Brilliant II SYBR® Green QRT-PCR Master Mix (Agilent), RT/RNase block enzyme, 200 nM of each primer and 300 ng of RNA solution. The qRT-PCR was carried out using the Mx3000P QPCR System machine (Agilent) at 42 °C for 30 min, 55 °C for 30 min, initial denaturation at 95 °C for 10 min and 40 cycles of 95 °C for 30 s, 60 °C for 30 s and 72 °C for 30 s. To analyze the dissociation curve, a thermal profile consisting of 95 °C for 1 min, then ramped down to 55 °C (0.5 °C/s) for 45 s and ramped up to 95 °C was used.

### 2.12. Molecular Docking to Determine the Interaction between the Nanobodies and the HCV NS3/4A

Computerized intermolecular docking was used for determining the interactions between the HCV protease specific-nanobodies with the NS3/4A molecule. For modeling of the JFH-1 NS3/4A, the program MODELLER based webserver, ModBase: Database of Comparative Protein Structure Models [30] was used. The model selected from the server was based on sequence homology with the templates from the database. The sequence of the JFH NS3/4A showed the highest homology with PDB 3O8B. The quality criteria of the model were validated by VADAR version 1.8 [31]. The details are given in the Supplemental data 1. The amino acid sequences of the nanobodies were subjected to homology modeling by the I-TASSER server service [32]. The predicted models derived from the I-TASSER were subsequently refined by using the high-resolution protein structure refinement, ModRefiner [33] and the fragment-guided molecular dynamics (FG-MD) simulation [34]. The local geometric and physical quality of the predicted 3D structures was improved by making them become closer to their native state. The NS3/4A and the nanobody models were subjected to HADDOCK server [35] for molecular docking. The largest cluster size with minimal local energy and a near-native state of the protein conformations was chosen for each docking. The pictures of top five clusters are shown in Supplemental data 2. The protein structure models and the molecular interactions were built and visualized by using the Pymol software (The PyMOL Molecular Graphics System, Version 1.3r1 edu, Schrodinger, LLC, NY, USA).

### 2.13. Statistical Analysis

Means and standard deviations of three independent experiments were used for comparison between tests and controls. *p* < 0.05 of unpaired *t*-test was considered significantly different.

## 3. Results

### 3.1. Recombinant NS3/4A Protease

**Figure 1:**
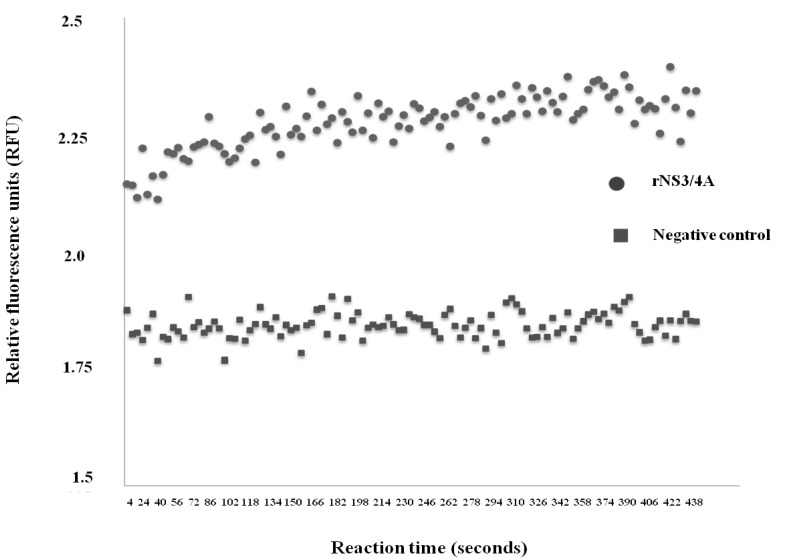
Protease activity of the recombinant rNS3/4A protein as determined by fluorescence resonance energy transfer (FRET) assay. The rNS3/4A cleaved the fluorogenic substrate, DE-D(EDANS)-EE-Abu-ψ-[COO]-AS-K-( DABCYL), and released the fluorescent signals from the separated substrate peptides, shown as relative fluorescence units (RFU) per second.

Amplicon of gene sequence coding for the rNS3/4A (710 bp) was successfully amplified from the cDNA synthesized from the HCV genotype 3a genomic RNA. The transformed *E. coli* clone carrying the recombinant plasmid with the inserted NS3/4A DNA produced the rNS3/4A after growing under the IPTG induction condition. The protein was contained in both soluble and insoluble fractions of the bacteria. The rNS3/4A in the insoluble fraction was purified under denaturing condition. The protease activity of the purified rNS3/4A was determined by FRET assay. The recombinant fusion protein cleaved the fluorogenic substrate, DE-D(Edans)-EE-Abu-ψ-[COO]-AS-K-(Dabcyl), and released of the fluorescent signals from the separated substrate peptides, shown as relative fluorescence units (RFU) in Figure 1.

### 3.2. HCV Protease Specific Nanobodies and Their Characteristics

**Figure 2:**
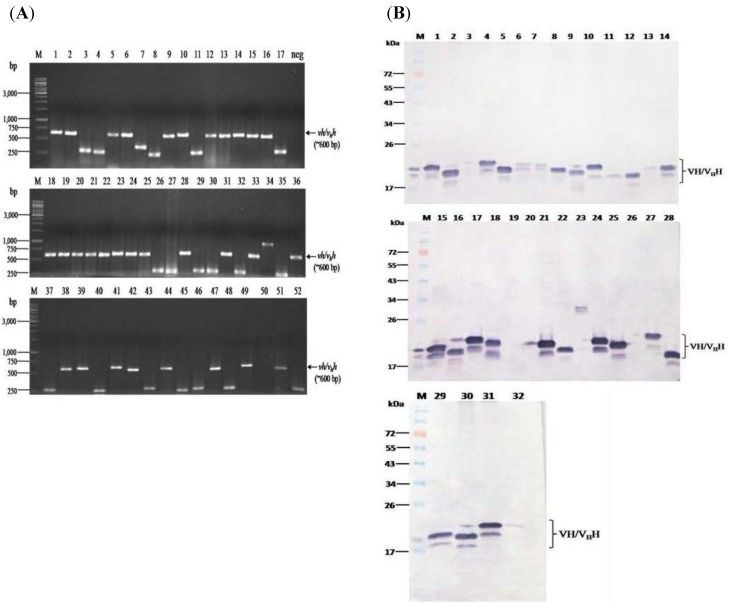
Amplicons of humanized-*vhs/v_h_hs* in the HB2151 *E. coli* clones transfected with the NS3/4A-bound phages and the VHs/V_H_Hs contained in the *E. coli* lysates revealed by Western blot analysis. (**A**) Results of PCR amplification of *vhs/v_h_hs* using 52 randomly picked phage transformed HB2151 *E. coli* colonies as templates and *R1* and *R2* primers. The expected size of *vh/v_h_h* sequences including flanking region of phagemid was ~600 bp (arrows). Lane M of all three blocks, GeneRuler^TM^ 1 kb DNA ladder; lanes 1, 2, 5, 6, 9, 10, 12–16, 18–25, 28, 31, 33, 36, 38, 39, 41, 42, 44, 47, 49 and 51 were 32 *E. coli* clones that were positive for the *vh/v_h_h* amplicons; lane neg, negative control (PCR mixture without DNA template). Numbers at the left of all blocks are DNA sizes in base pairs (bp); (**B**) Western blot results for detecting the VHs/V_H_Hs in lysates of the 32 *E. coli* clones that carried the *vhs/v_h_hs*. Lane M, Pre-stained standard protein ladder; lanes 1–2, 4–18, 21–22, 24–25 and 27–32 show VHs/V_H_Hs (~19–22 kDa) expressed from 28 clones; lane 3, 19, 23 and 26 were lysates of *vh/v_h_h-*positive *E. coli* clones that did not express VHs/V_H_Hs. Numbers at the left of all blocks are protein molecular sizes in kDa.

The rNS3/4A protein with the inherent HCV protease activity was used as the antigen in the panning process to select phage clones that bound to the protein from the humanized-camel VH/V_H_H phage display library. From 52 randomly picked phage transformed *E. coli* colonies grown on the LB-AG plate, 32 colonies harbored the *vh/v_h_h* sequences (~600 bp) as determined by PCR (Figure 2A). Among them, 28 cloned produced soluble VHs/V_H_Hs (~19–22 kDa) detectable by Western blotting usingrabbit anti-E tag antibody as the antibody detection reagent (Figure 2B).

**Figure 3:**
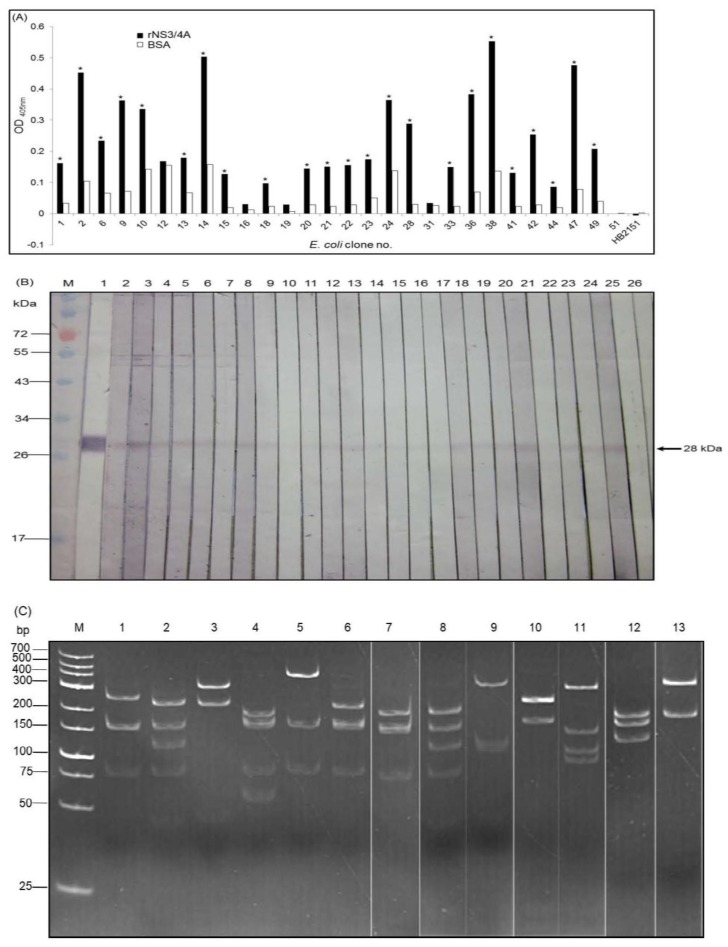
Binding of the VHs/V_H_Hs expressed from the 28 *vh/v_h_h-*positive *E. coli* clones to the rNS3/4A and restriction fragment length polymorphism (RFLP) of the *vhs/v_h_hs* of the antigen specific nanobodies. (**A**) Indirect ELISA results. Nanobodies from 23 of the 28 *E. coli* clones gave significant OD_405nm_ ELISA signal to the rNS3/4A above the control antigen (BSA). They were clone nos. 1, 2, 6, 9, 10, 13–15, 18, 20–24, 28, 33, 36, 38, 41, 42, 44, 47 and 49. Normal *E. coli* lysate (HB2151) was used as the background control; (**B**) Western blotting results. The VHs/V_H_Hs of the 23 clones also bound specifically the SDS-PAGE separated-rNS3/4A(lanes 2–24). Lane M, Protein molecular mass marker. Lane 1, SDS-PAGE separated-rNS3/4A probed with mouse anti-6× His tag which served as positive control; (**C**) DNA banding patterns (RFLP) of *vhs/v_h_hs* from the 23 *E. coli* clones which revealed 13 different patterns. Lane 1, pattern 1 (clone no. 1); lane 2, pattern 2 of clone nos. 2, 14, 22 and 44; lane 3, pattern 3 of clone nos. 6, 21 and 36; lane 4, pattern 4 of clone no. 9; lane 5, pattern 5 of clone nos. 10, 28 and 49; lane 6, pattern 6 of clone nos. 13 and 47; lane 7, pattern 7 of clone no. 15; lanes 8–13, pattern 8–13 of clone nos. 18, 20, 24, 28, 33 and 41, respectively. Lane M, GeneRuler^TM^ 1 kb DNA ladder.

Lysates of the 28 *E. coli* clones were subjected to indirect ELISA and the nanobodies in 23 lysates (clone nos. 1, 2, 6, 9, 10, 13–15, 18, 20–24, 28, 33, 36, 38, 41, 42, 44, 47 and 49) bound to the rNS3/4A and gave significant OD_405nm_ above the control antigen (BSA) (more than two times higher) (Figure 3A). The VHs/V_H_Hs of the 23 clones also bound specifically the SDS-PAGE separated-rNS3/4A (arrow) in the Western blot analysis (Figure 3B).

Restriction fragment length polymorphism (RFLP) of the *vh/v_h_h* sequences of the 23 clones revealed 13 different DNA banding patterns (Figure 3C). Clone no. 1 showed pattern 1; clone nos. 2, 14, 22 and 44 had pattern 2; clone nos. 6, 21 and 36 had pattern 3; clone no. 9 had pattern 4; clone nos. 10, 28 and 49 had pattern 5; clone nos. 13 and 47 had pattern 6 and clone no. 15 had pattern 7. Clone nos. 18, 20, 24, 28, 33 and 41 had patterns 8–13, respectively.

The nucleotides of *vhs/v_h_hs* of the 23 clones were sequenced and the deduced amino acids of their CDRs and FRs were determined by using IMGT information system. The amino acid sequences of clone nos. 1, 10 and 49; 20 and 21; and 6 and 36 were identical. The amino acid sequences of clone nos. 24, 28 and 41 which their coding sequences had different DNA banding patterns showed the characteristic amino acid tetrads of the heavy chain antibody V_H_H, *i.e*., Y/S/F42, E49, R50 and F/G52, in the FR2. These three clones were selected for further study.

### 3.3. Cell Penetrable V_H_Hs

**Figure 4:**
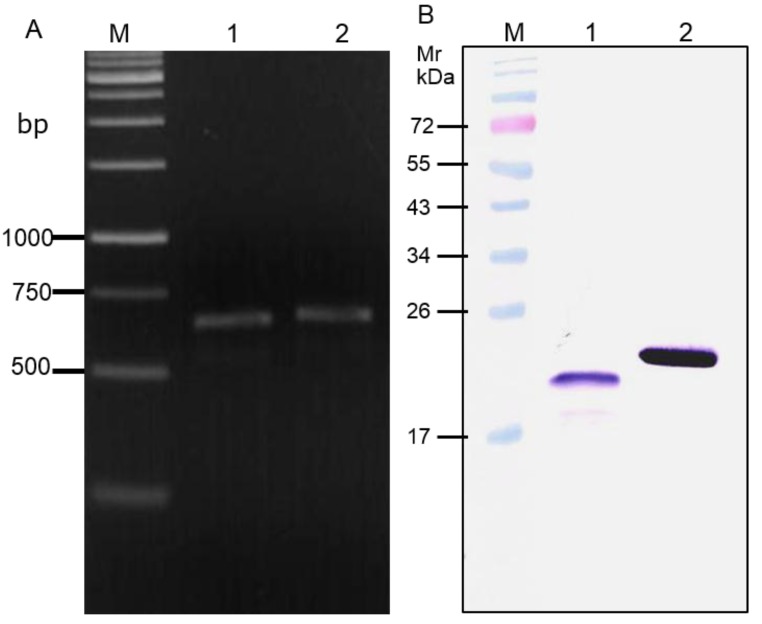
Preparation of cell penetrable nanobody (transbody). (**A**) Amplicons of the gene sequences coding for V_H_H24 and PEN-V_H_H24 (lanes 1 and 2, respectively). The latter is slightly larger than the former. Lane M, molecular size DNA ladder. Numbers at the left are DNA sizes in bp; (**B**) V_H_H24 and PEN-V_H_H24 (lanes 1 and 2, respectively). Lane M, Pre-stained molecular weight protein marker. Numbers at the left are protein relative molecular masses (Mr) or protein molecular masses in kDa.

The gene sequences coding for the V_H_H24 (accession no. KR045613), V_H_H28 (accession no. KR045614) and V_H_H41 (accession no. KR045615) were inserted into *pen-*pET23b+ plasmid immediately downstream of the PEN coding sequence. Figure 4A shows representative amplicons of the gene sequences coding for the PEN-nanobodies (*pen-v_h_h24*) and the nanobody (*v_h_h24*). The constructed plasmids were introduced into BL21 (DE3) *E. coli.* The respective PEN-V_H_Hs were produced as inclusions in the *E. coli* insoluble fractions. The PEN-V_H_Hs were obtained after purification. Representatives of the PEN-V_H_Hs and V_H_Hs counterparts are shown in Figure 4B.

### 3.4. Cellular Internalization and Cytotoxicity of the PEN-V_H_Hs

**Figure 5:**
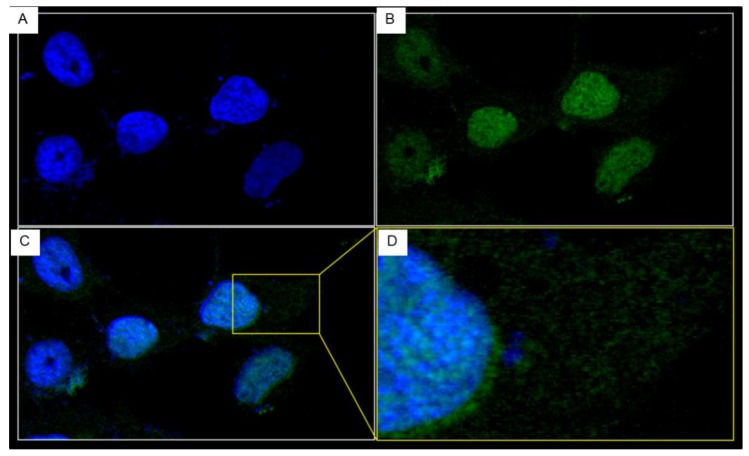
Intracellular localization of the PEN-V_H_H24 revealed by confocal microscopy. Huh7 cells treated with 10 μg of PEN-V_H_H24 for 24 h were washed, fixed with cold methanol, permeated with 1% Triton X-100 before adding with mouse anti-6× His Tag and donkey anti-mouse immunoglobulin (DyLight_488), respectively. (**A**) DAPI was used to stain the cell nuclei (blue); (**B**) The penetratin linked-nanobodies appear in green in the cells; (**C**) Merged A and B; (**D**) Enlarged square area of (**C**) showing localization of the PEN-V_H_H24 in both nucleus and cytoplasm.

The Huh7 cells incubated with 10 μg of the PEN-V_H_Hs were stained for nuclei by DAPI and PEN-V_H_Hs by using mouse anti-6× His tag (1:1000) and donkey anti-mouse immunoglobulin (DyLight® 488) before subjecting to a confocal microscopy. It was found that the penetratin linked-nanobodies readily entered the mammalian cells of which the representatives are shown in Figure 5A–D. The amounts of individual PEN-V_H_Hs in the cells were >85% of the incubating amount (data not shown).

The Huh7 cells cultured in the medium containing 25 μg of PEN-V_H_Hs for 24 h had no significant release of the LDH into the culture fluids compared to the non-treated cell control (data not shown) indicating negligible toxicity of the transbodies to the human cells.

### 3.5. Inhibition of the HCV Replication by the Protease Specific-Transbodies

**Figure 6:**
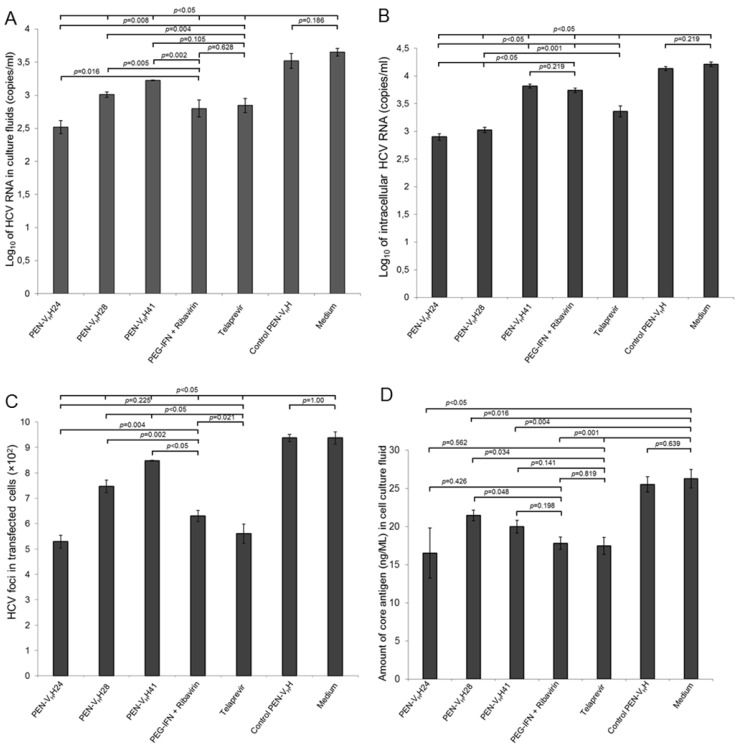
The numbers of the hepatitis C virus (HCV) 5’ UTR RNA, infectious particles and core antigen in the culture supernatants and/or inside the transfected cells treated with the protease specific-transbodies and controls. (**A**) and (**B**) Amounts of HCV RNA in the culture supernatants and inside the transfected Huh7 cells treated with the HCV protease specific-transbodies (PEN-V_H_H24, PEN-V_H_H28 and PEN-V_H_H41), PEG-IFN-α + ribavirin, telaprevir, irrelevant (control) transbody and medium alone, respectively; (**C**) The numbers of the HCV foci in the transfected cells treated with the HCV protease specific-transbodies and controls as determined by foci assay; (**D**) Amounts of the HCV core antigen in culture supernatants of HCV RNA transfected cells after receiving different treatments. *p* < 0.05 is significantly different.

The JFH-1 RNA transfected-Huh7 cells cultured in the medium containing 25 µg of PEN-V_H_H24, PEN-V_H_H28, PEN-V_H_H41, 100 units of PEG-IFN-α + 50 nM ribavirin, and 0.175 μM telaprevir had significantly less HCV 5’ UTR RNA in the culture supernatants (Figure 6A) and inside the cells (Figure 6B) than the transfected cells cultured in the medium alone (negative inhibition control) and the cells treated with control PEN-V_H_H (background inhibition control) (*p* < 0.05). Efficacy of the PEN-V_H_H24 was slightly higher than the PEG-IFN-α + ribavirin and telaprevir.

**Figure 7:**
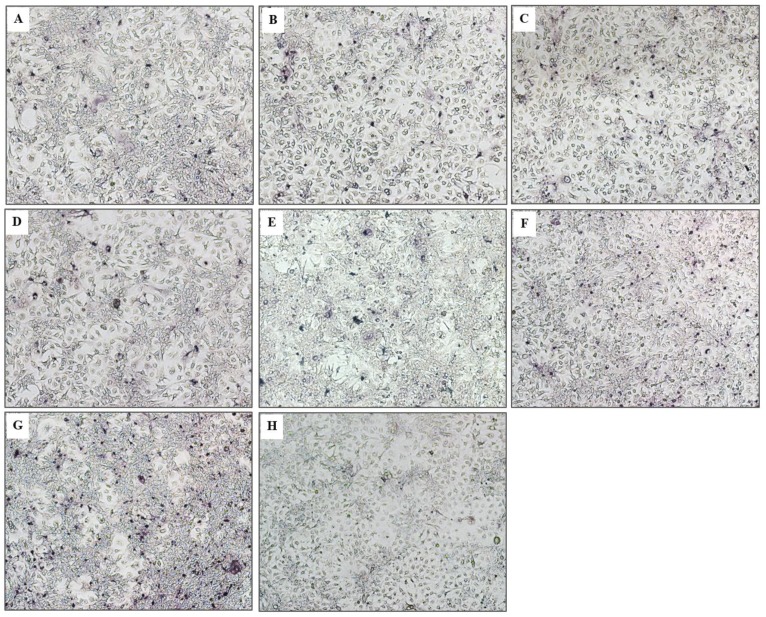
HCV foci in Huh 7 cells treated with cell penetrable V_H_Hs and controls. Core antigen in the foci was detected by mouse anti-HCV core antibody, goat anti-mouse immunoglobulin-AP conjugate and substrate. (**A**)**–**(**E**) The numbers of foci in the transfected cell treated with PEN-V_H_H24, PEN-V_H_H28, PEN-V_H_H41, PEG-IFN-α + ribavirin and telaprevir, respectively, which were significantly less than the transfected cells treated with control PEN-V_H_H (**F**) and medium alone (**G**). (**H**) Non-infected Huh7 cells.

The numbers of the HCV foci in the transfected cells treated with the transbodies (PEN-V_H_H24, PEN-V_H_H28 and PEN-V_H_H41), PEG-IFN-α + ribavirin and telaprevir determined by the foci assay were significantly less than the transfected cells cultured in the medium alone or control PEN-V_H_H (*p* < 0.05) (Figure 6C). Efficacy of the transbodies and the controls were PEN-V_H_H24 = telaprevir > PEG-IFN-α + ribavirin > PEN-V_H_H41 > PEN-V_H_H28 > control-PEN-V_H_H = medium alone. Likewise, the amounts of HCV core antigen in the cell culture supernatants of the transfected cells treated with the viral protease specific transbodies, PEG-IFN-α + ribavirin and telaprevir were also less than the transfected control and the cells treated with the control transbody (Figure 6D). Appearances of the HCV foci inside the transfected Huh7 monolayer of all treatments are shown in Figure 7.

### 3.6. Response of the HCV Transfected-Cells to Treatment with Protease Specific-Transbodies

**Figure 8:**
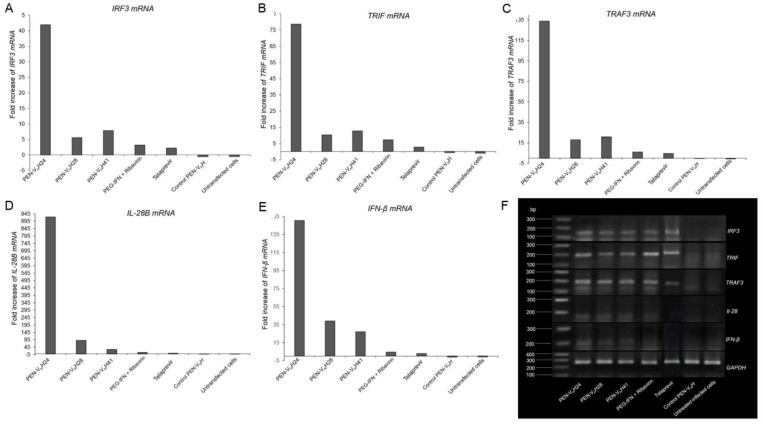
Expressions of innate immune response genes including *IRF-3*, *TRIF*, *TRAF-3*, *IL-28B* and *IFN-β* of the JFH-1 RNA transfected human hepatic (Huh7) cells that received various treatments. (**A**–**E**) Fold increase of the expressions of the genes of the infected cells treated with PEN-V_H_Hs, control PEN-V_H_H, PEG-IFN-α + ribavirin, and telaprevir, respectively, in comparison with the control transfected cells cultured in the medium alone. There were significant increases of the mRNAs of all innate immune response genes in the transfected cells treated with the PEN-V_H_H24, PEN-V_H_H28, PEN-V_H_H41, PEG-IFN-α + ribavirin and telaprevir compared with the transfected cells cultured in the medium alone, treated with control transbody and untransfected cells (*p* < 0.05). The PEN-V_H_H24 showed the highest efficacy; (**F**) Agarose gel electrophoretic appearances of ethidium bromide stained-amplicons of *IRF-3*, *TRIF*, *TRAF-3*, *IL-28B* and *IFN-β* mRNAs extracted from the transfected cells that received different treatments and the untransfected cells. The house-keeping GAPDH gene was used for normalization. The agarose gel electrophoresis is not as sensitive as the qRT-PCR; the bands of *IFN-β* in the PEG-IFN-β + ribavirin and telaprevir treated cells could not be seen in the gel.

Fold increase of the mRNA expressions of the innate immune response genes, *i.e*., *IRF3*, *TRIF*, *TRAF3*, *IL-28B* and *IFN-β*, of the JFH-1 RNA transfected Huh7 cells after treating with the protease specific-transbodies, control PEN-V_H_H, PEG-IFN-α + ribavirin, telaprevir and untreated transfected cells as determined by qRT-PCR are shown in Table 1 and Figure 8A–E, respectively. There were significant increases of the mRNAs of all studied innate immune response genes in the transfected cells treated with the PEN-V_H_H24, PEN-V_H_H28 and PEN-V_H_H41, PEG-IFN-α + ribavirin and telaprevir compared with the transfected cells without any treatment, treated with control transbody or the untransfected cells (*p* < 0.05). Results of agarose gel electrophoresis of the qRT-PCR amplicons of the innate immune response genes of all treatments are shown in Figure 8F using the GAPDH gene for normalization.

### 3.7. Computerized Binding of the Nanobodies to the HCV NS3/4A Protease

The HCV genotype 3a NS3-4A was modeled by MODELLER software while the V_H_H24, V_H_H28 and V_H_H41 were predicted by the I-TASSER. The most accurate data were extracted from the best scores for estimating the modeled quality. The molecular docking between the HCV NS3/4A with the V_H_H24, V_H_H28 and V_H_H41 performed by the HADDOCK server were predicted in four separate modes including, balance, electrostatic-favored, hydrophobic-favored and Van der Waals + Electrostatic. Different clusters were provided by the server and the ones with the lowest energy scores were collected while the other clusters are provided in the Supplemental data 2. The lowest energy scores for the complexes of the HCV NS3/4A with the V_H_H24, V_H_H28 and V_H_H41 were −476.7 ± 39.8, −716.0 ± 40.9 and −546.9 ± 33.3 kcal/mol, respectively.

**Figure 9:**
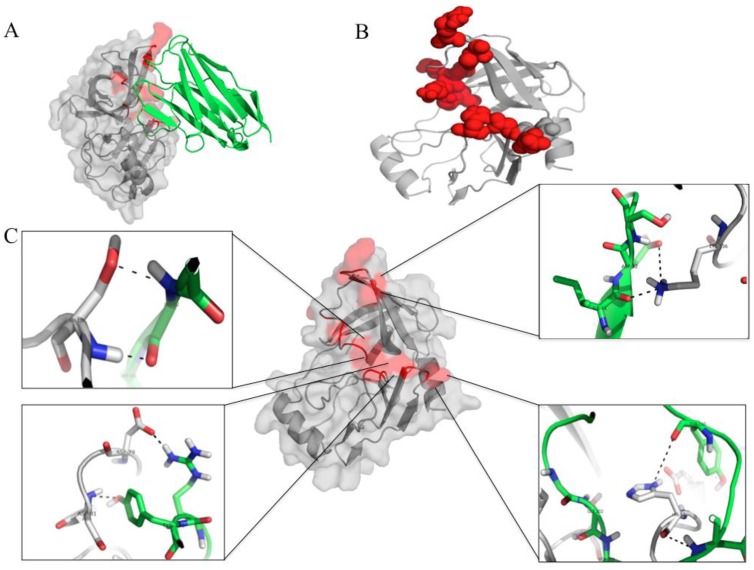
The molecular interaction between the HCV NS3/4A protease and the protease specific-V_H_H24. (**A**) and (**B**) The nanobody (green in A) uses CDRs 2 and 3 in binding to spatially juxtaposed areas of the folded protease molecule (red). (**C**) Details of the interactive amino acid residues of the nanobody (green) and the NS3 protein (grey) (see also Table 1A).

According to the docking outputs, the V_H_H24 was found to bind to several residues in three juxtaposed regions of the NS3/4A protein by using CDRs 2 and 3 (Figure 9A–C). Among the bound residues were two residues of the catalytic triad (H57/H1089 and D81/D1113 of the HCV NS3/polyprotein) and one residue of the oxyanion loop (K136/K1168 of the NS3/polyprotein). Details of the interactive residues between the two proteins are given in Figure 9C and Table 1A.

**Figure 10:**
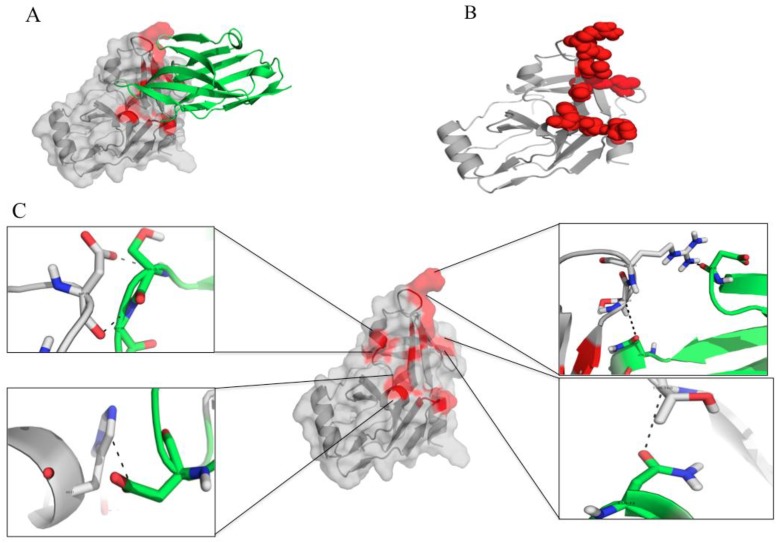
The molecular interaction between the HCV NS3/4A protease and the protease specific-V_H_H28. (**A**) and (**B**) The nanobody (green in A) protrudes CDRs 2 and 3 and FR2 to bind to three areas of the folded protease molecule (red). (**C**) Details of the interactive residues of the V_H_H28 (green) and the target NS3 protein (grey) (see also Table 1B).

Binding of the V_H_H28 to the HCV NS3/4A are illustrated in Figure 10A–C. The V_H_H28 interacted with many residues of the HCV protein. Among them were H57/H1089 and D81/D1113 of the catalytic triads and L135 of the S1 site. Figure 10C and Table 1B give details on the interactive residues of NS3/polyprotein of HCV genotype 3a with the V_H_H28.

Table 1Residues and motives of the HCV NS3/4A/polyprotein bound by the residues and domains of the NS3 specific humanized-V_H_Hs. (**A**) Humanized-V_H_H24; (**B**) Humanized-V_H_H28; (**C**) Humanized-V_H_H41.

**Table:** (**A**)

	HCV Protein		Humanized-V_H_H24		Intermolecular Bond
NS3 Residue	Polyprotein Residue	Motif	Amino Acid	Domain	
Y56	Y1088	α -helix a	D107	CDR3	H bond
H57	H1089	α-helix a (catalytic triad)	Y110	CDR3	H bond
D81	D1113	Between β-E1 and β-F1 (catalytic triad)	Y110	CDR3	H-bond
A131	A1163	α-helix b	I51	CDR2	Hydrophobic
K136	K1168	Near α-helix b (oxyanion loop)	D50	CDR2	H bond

**Table:** (**B**)

	HCV Protein		Humanized-V_H_H28		Intermolecular Bond
NS3 Residue	Polyprotein Residue	Motif	Amino Acid	Domain	
H57	H1089	α-helix a (catalytic triad)	D101	CDR3	H-bond
S61	S1093	Between α-helix a and β-E1	D54	CDR2	H-bond
D81	D1113	Between α-helix a and β-E1	Y102	CDR3	H-bond
N123	N1155	β-C2	T73	FR2	H bond
L135	L1167	S1 pocket	W103	CDR3	Hydrohobic

**Table:** (**C**)

	HCV protein		Humanized-V_H_H41		Intermolecular Bond
NS3 Residue	Polyprotein Residue	Motif	Amino Acid	Domain	
Q9	Q1041	Before α-helix a	N115	CDR3	H-bond
R11	R1043	α-helix a	R114	CDR3	Salt bridge
L13	L1045	α-helix a	W119	FR4	Hydrophobic
P131	P1163	α-helix b	N73	FR3	H bond
L135	L1167	S1 pocket	Q3	FR1	H bond
K136	K1168	Near α-helix b (oxyanion loop)	Y26	CDR1	H-bond

**Figure 11:**
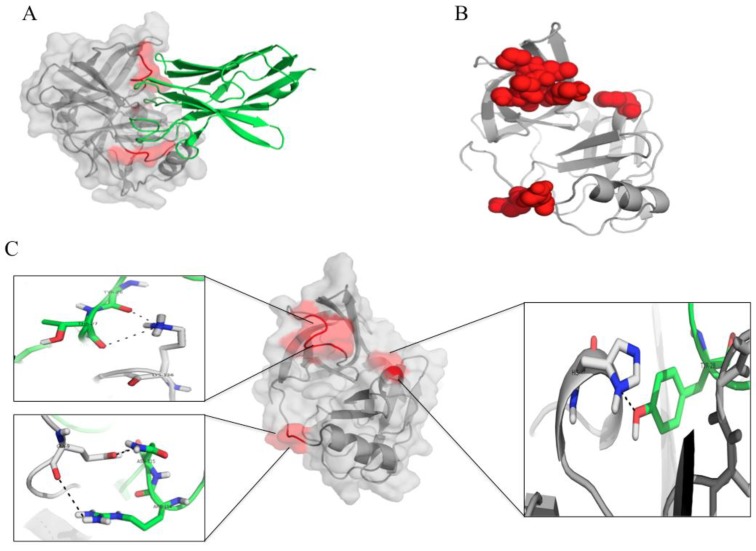
The interactive residues between the HCV NS3/4A protease and the protease specific-V_H_H41. (**A**) and (**B**) The nanobody (green in A) uses CDRs 1 and 3 and FRs 1, 3 and 4 to bind to juxtaposed areas of the folded protease molecule (red); (**C**) Details of the interactive residues between the nanobody (green) and the target (grey) (see also Table 1C).

Molecular interaction between the V_H_H41 and the HCV NS3/4A are illustrated in Figure 11A–C. Details of the interaction of the two parties are given in Table 1C. The amino acids of the NS3/polyprotein bound by the V_H_H41 were Q9, R11 and L13 in the N-terminal, L135 of the S1 pocket and K136/K1168 of the oxyanion loop of the protease. The important regions of the NS3/4A that the V_H_Hs bind are shown in Supplemental data 3.

## 4. Discussion

Anti-HCV agents that are highly and broadly effective, able to cope better with the virus mutation and well tolerated by the treated patients are needed. The hepatitis C treatment strategy that we are proposing consists of a combination of ready to use, engineered, cell penetrable small human/humanized-antibodies that are specific to multiple epitopes of the HCV key enzymes including helicase [24] polymerase [25,27] and protease (present study).

Because of their small sizes (15–25 kDa), high tissue penetrating efficacy and relatively high thermal stability, the V_H_Hs are attractive enzyme and toxin inhibitors [21,36,37]. The nanobodies have relatively long CDRs and can recognize the conformational structure within the pocket of the target enzyme active site. They may insert CDRs into the catalytic groove and blocked directly the enzymatic activity which the antigen-binding fragments (VH-VL) of the conventional antibody molecules cannot do so [21,38]. In the present study, therefore, cell penetrable humanized-V_H_Hs that bound specifically to and blocked the activity of the HCV protease leading to inhibition of the HCV replication were produced by using the previously established humanized-VH/V_H_H phage display library [21] with the purpose of testing them further in clinical trials for human use. The NS3 protease is an attractive target for the development of anti-HCV therapy, as not only it is responsible for processing of the viral polyprotein during replication, but also the protein is pivotal for the viral evasion of the host immunity.

In this study, recombinant NS3/4A protein that consisted of the N-terminal 180 residues of the NS3 fused via a peptide linker to residues 21–32 of the NS4A was produced in *E. coli*. It has been known that the V23, G27, R28, and in particular I25 and I29 residues are critical for protease cofactor function of the NS4A [37]. The NS4A peptide-bound NS3 had an increased substrate turnover rate (*K_cat_*) compared with the NS3 alone [39]. The so-produced purified recombinant NS3/4A protein readily and specifically cleaved a fluorogenic synthetic substrate containing Asp at the P6, aminobutyric acid (substitution of Cys) at P1 and Ala at P1’ [DE-D(Edans)-EE-Abu-ψ-(COO)-AS-K-(Dabcyl)] indicating that the *E. coli* expressed protein was properly folded. Therefore, the enzymatic active protein was used as the antigen in the biopanning for selecting the protein bound phage particles from the humanized-VH/V_H_H phage display library. After linking the V_H_Hs to a cell penetrating peptide, penetratin (PEN), the PEN-V_H_Hs readily entered the mammalian cells but did not cause significant release of LDH from the cells indicating their cellular compatibility.

The cell penetrable nanobodies (transbodies) expressed by three transformed *E. coli* clones (V_H_H24, V_H_H28 and V_H_H41) could inhibit replication of the heterologous JFH-1 RNA in the human hepatic (Huh7) cells transfected with the RNA as shown by reduction of the HCV 5’ UTR RNA inside the cells and in the culture fluids, reduction of HCV foci in the treated transfected cells and reduction of core antigen in the culture supernatants; indicating a cross-neutralizing activity of the protease specific-transbodies. The activity of the V_H_H24 was as effective as the PEG-IFN-α + ribavirin and telaprevir or even higher.

To assess the tentative mechanisms of the V_H_H-mediated inhibition of the HCV replication, computerized homology modeling and molecular docking were used to determine the regions and residues of the NS3/4A protease bound by the nanobodies. The V_H_H24 which showed the most efficient inhibition of the HCV replication was found to use CDR2 and CDR3 to dock on several conserved residues of the NS3/4A protein. Among them were H57 and D81 of the NS3 N-terminal subdomain (H1089 and D1113 of the polyprotein, respectively) which are important for spatially forming the protease catalytic triads [40]. From previous study, substitution of any of the catalytic triad residues (H57, D81 and S139) abolished the ability of the NS3/4A protease to cleave the NS3/NS4A, NS4A/NS4B, NS4B/NS5A and NS5A/NS5B junctions which led to interference of the viral replication process [41,42]. The V_H_H24 also interacted with the K136 in the β-E2 motif which forms part of the protease oxyanion hole [40]. Taken altogether, it is likely that the V_H_H24 transbody inhibited the HCV replication by interfering with the protease catalytic activity and the oxyanion loop function.

From the docking, the V_H_H28 transbody used CDR2 and CDR3 to bind to H57 and D81 of the NS3 catalytic triad and L135. The L135 of the HCV NS3 is one of the three hydrophobic residues that form the S1 pocket which favors interaction with the substrate P1 side chain [40,43]. The V_H_H41 bound also to the L135. Binding of the V_H_H28 and V_H_H41 to the S1 pocket might contribute also to the alteration of the NS3/4A activity leading to the reduction of the HCV replication. Besides, the V_H_H41 used CDR3 and FR4 to bind to Q9, R11 and L13 in the N-terminal 22 residues of the NS3 which involved in the interaction with the NS4 cofactor [44,45]. CDR1 of the V_H_H41 interacted also with the K136 of the oxyanion loop. The computerized modeling and intermolecular docking therefore shed some light on the mechanisms of HCV replication inhibition mediated by the V_H_H transbodies. The cell penetrable V_H_Hs produced in this study seem to intrigue several critical residues that are highly conserved and which never been reported in any of the protease inhibitor resistant mutants [46]. Therefore, it is likely that the V_H_H transbodies should be able to cope with the HCV mutations. They are likely also to be broadly and highly effective anti-HCV agents. Studies are needed to verify these speculations.

Pathogen-associated molecular patterns (PAMPs) of viruses, e.g., dsRNA, interact with host pattern recognition receptors (PRRs) such as Toll-like receptors (TLRs), RIG-1-like receptors and NOD-like receptors (NLRs) and consequently stimulate production of type 1 interferons (IFN-α, β and ω) and other anti-viral substances [47]. The HCV NS3/4A protease cleaves the key adapter protein, MAVs (CARDIF/IPS-1) that usually transmits signals from RIG-1 or MAD5 to downstream kinases (IKKε/IKKα/β/γ complex/TBK-1/MAPK), rendering refractoriness of the IRF3/7, NF-κB and AP-1 transcription factors and hence no innate interferon gene activation and the interferon production. The viral enzyme also cleaves TRIF which is another key adapter protein of the TLR3 signaling pathway and thus blocks the TLR3 mediated innate IFN production [48,49,50]. It is known that the type 1 interferons act in an autocrine and paracrine manner to stimulate the cells via JAK/STAT pathway leading to cellular production of several antiviral agents including PKR, 2,5 OAS and RNAi silencing system as well as MHC class I molecule for cytotoxic lymphocyte stimulation. Thus, by cleaving both MAVS and TRIF, the HCV readily mediates the host immunity evasion. About a decade ago, type III interferons including IFN-λ1, IFN- λ2 and IFN- λ3 encoded by *IL-29*, *IL-28A* and *IL-28B*, respectively were identified [51,52]. *IL-28* shares a similar activation pathway with the type I interferons and can be up-regulated by the HCV 3' UTR stimulation by means of the IRF3 and NF-κB dependent route. HCV NS3/4A cleaves TRIF and thus blocks the IL-28 production by inhibiting the type I interferon signaling pathway [53].

In conclusions, the PEN-V_H_Hs specific to NS3/4A protease produced in this study inhibited the HCV replication and readily rescued the host innate immune response that had been suppressed by the HCV. Although the V_H_Hs were selected from the naïve library and there is a possible risk of having polyreactivity which should be elucidated further, the overall data indicate that the transbodies particularly the PEN-V_H_H24, have high potential for developing into efficient immunotherapeutic agents for patients with the viral infection.

## Supplementary Files

Supplementary File 1
