# Supplementary material for: Humanized-VHH Transbodies that Inhibit HCV Protease and Replication
Source: Viruses. 2015 Apr 20;7(4):2030–56. doi: 10.3390/v7042030 (PMC4411689; doi:10.3390/v7042030)
Supplement: Supplementary File 1 [file viruses-07-02030-s001.pdf]

Supplementary Information

Supplemental data 1. The quality criteria of the NS3/4A models.

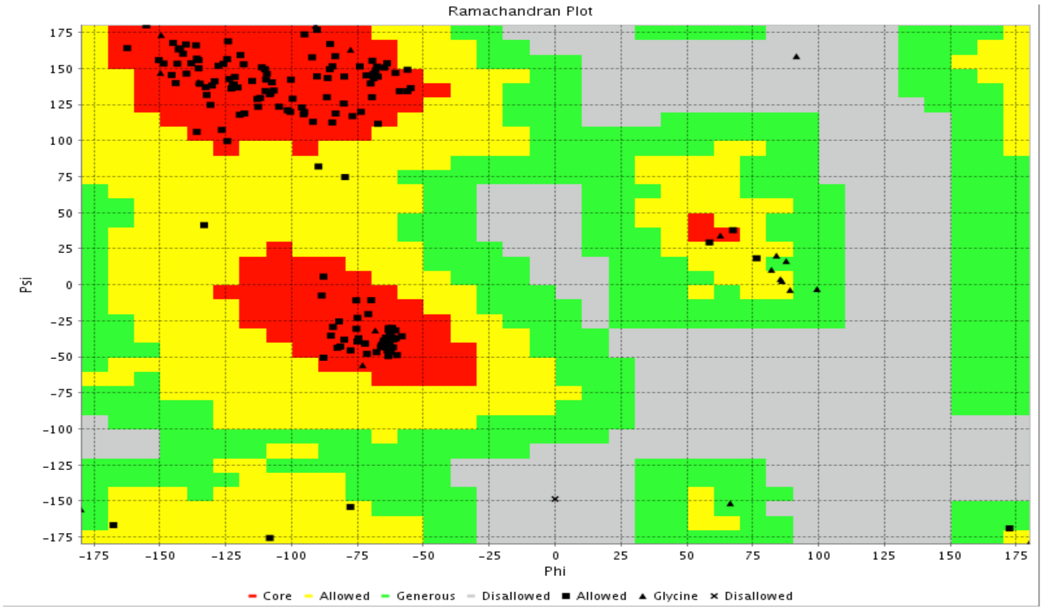

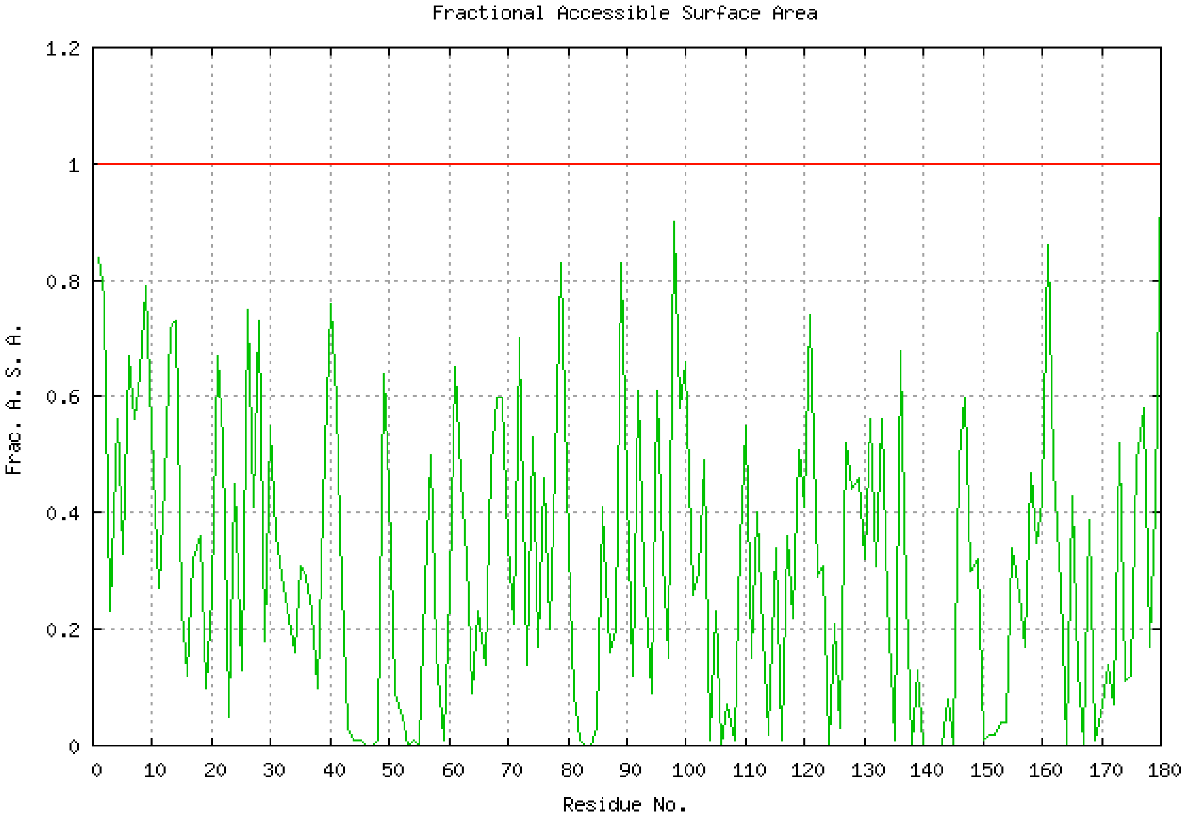

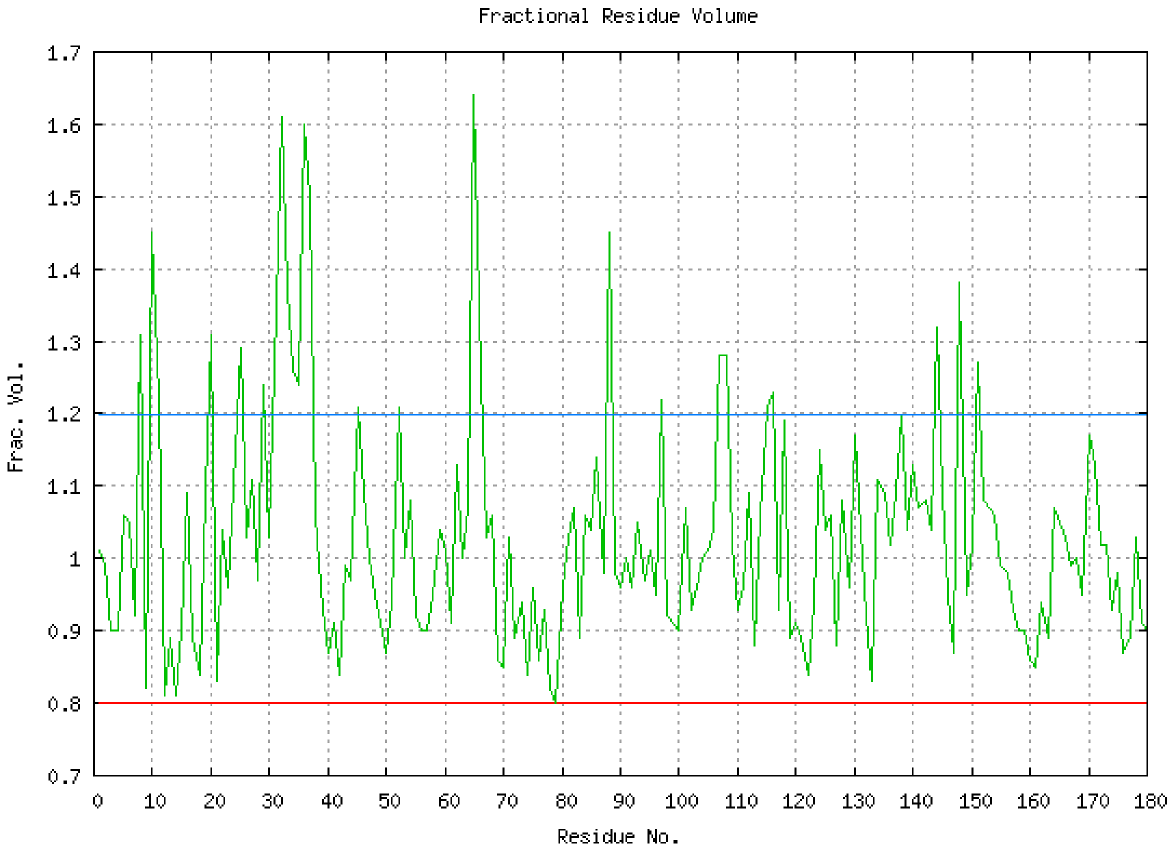
Fractional Residue Volume
Frac. Vol.
Residue No.
1.7
1.6
1.5
1.4
1.3
1.2
1.1
1
0.9
0.8
0.7
0
10
20
30
40
50
60
70
80
90
100
110
120
130
140
150
160
170
180

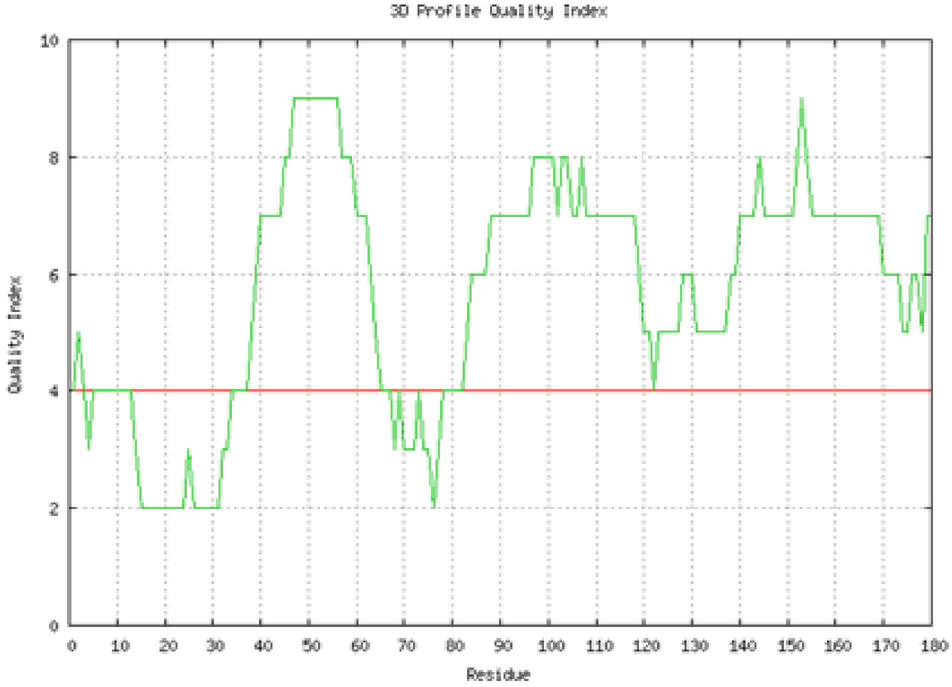
3D Profile Quality Index
Quality Index
Residue
10
8
6
4
2
0
0
10
20
30
40
50
60
70
80
90
100
110
120
130
140
150
160
170
180

Supplemental data 2. Top five clusters of interaction between the modeled NS3/4A and nanobody provided by the server

Graphical representations of the HADDOCK- clone V_HH24

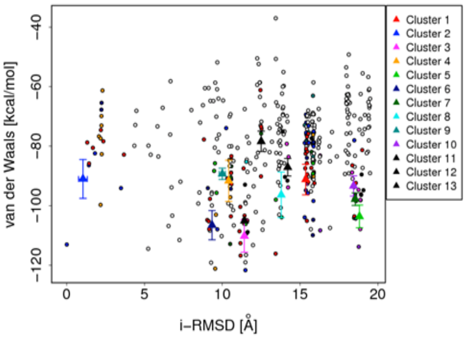

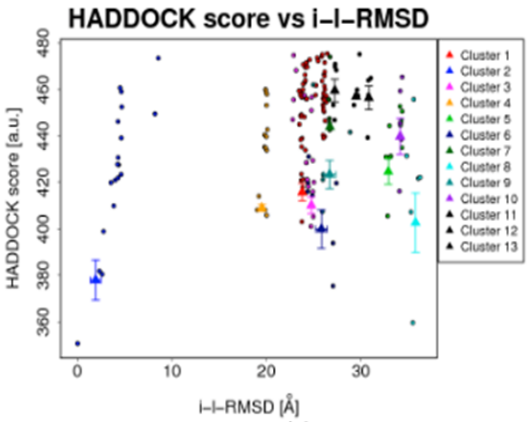

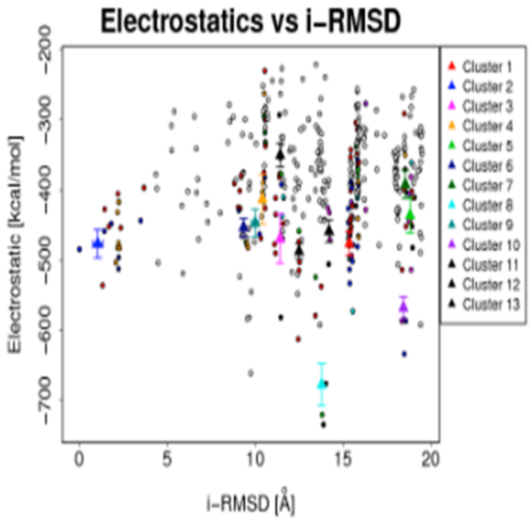

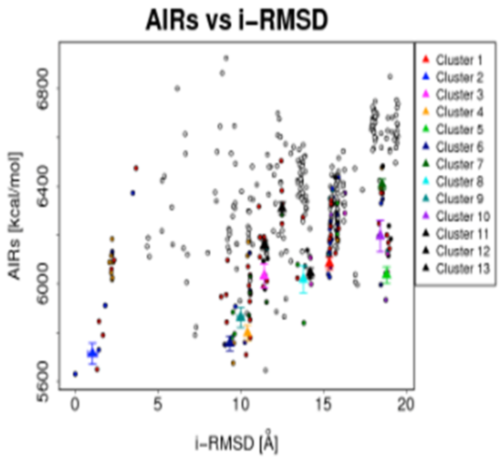

Clusters of NS3/4A- VH_H24

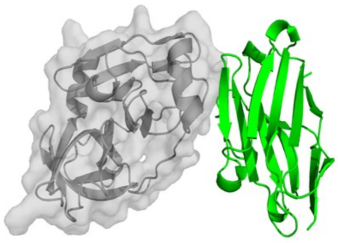
Cluster 1

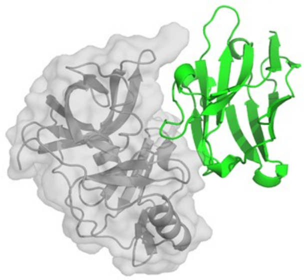
Cluster 6

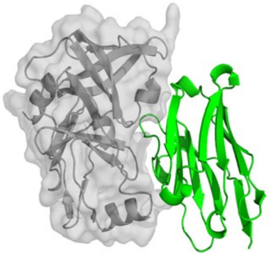
Cluster 8

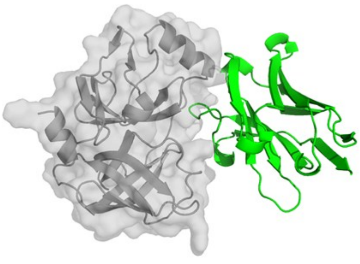
Cluster 4

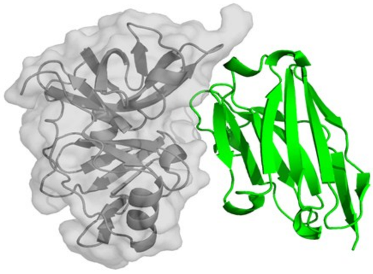
Cluster 3

Graphical representations of the HADDOCK- clone V_HH28

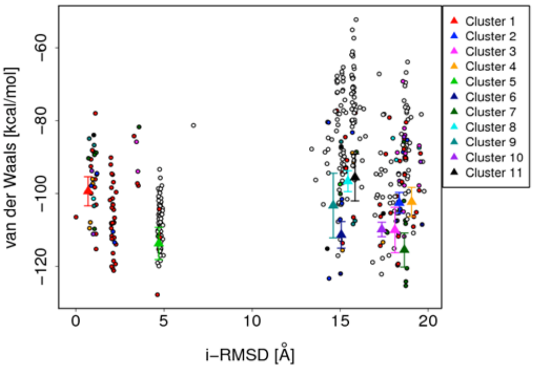

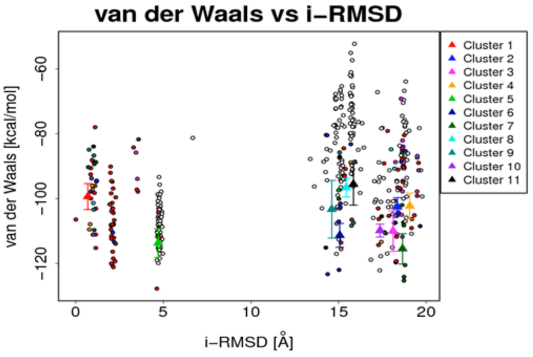

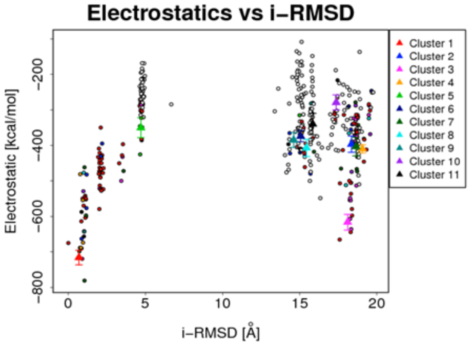

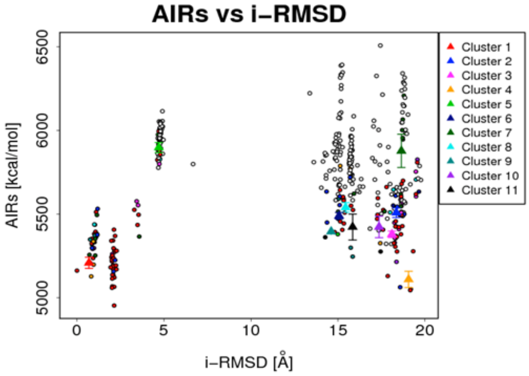

Clusters of NS3/4A-VH_H28

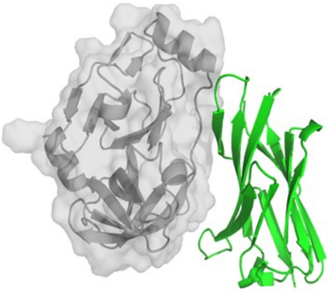

Cluster 3

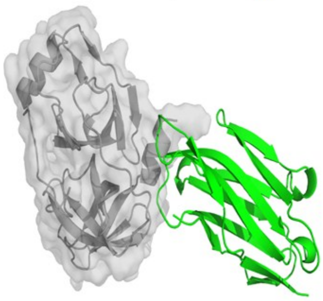

Cluster 4

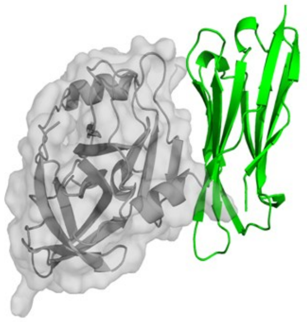

Cluster 2

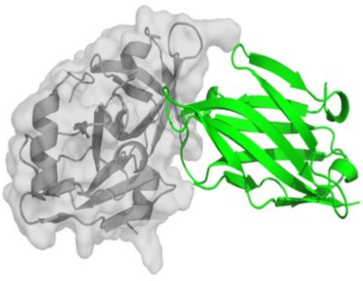

Cluster 8

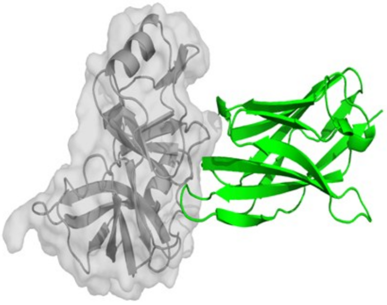

Cluster 6

Graphical representations of the HADDOCK- clone V_HH41

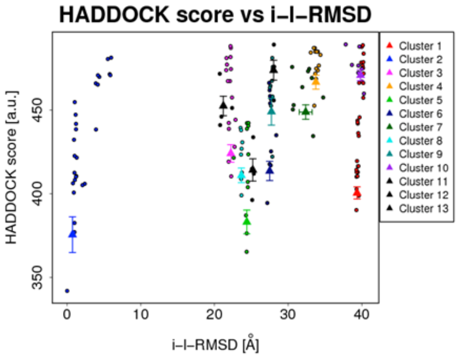

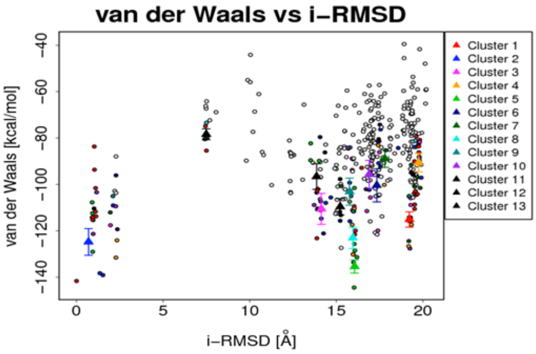

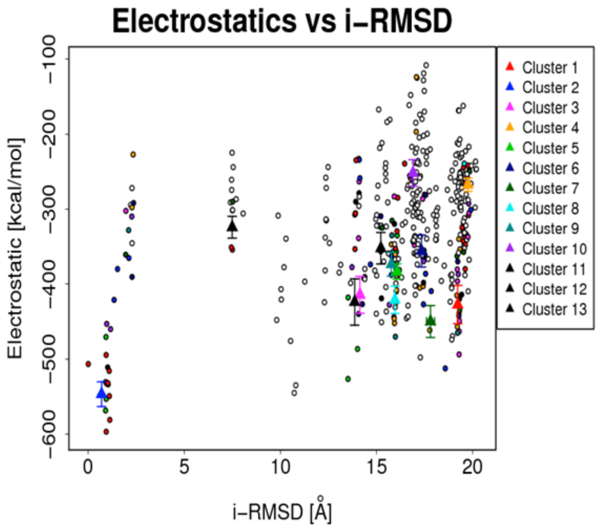

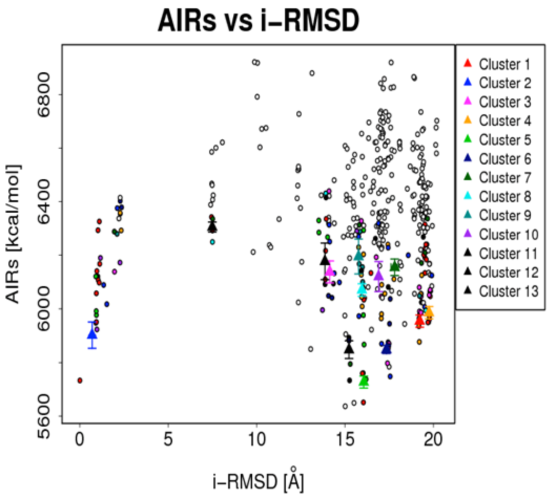

Clusters of NS3/4A-VH_H41

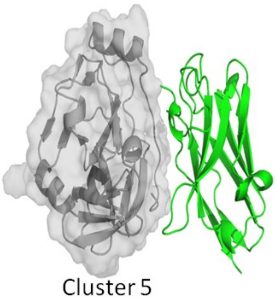

Cluster 5

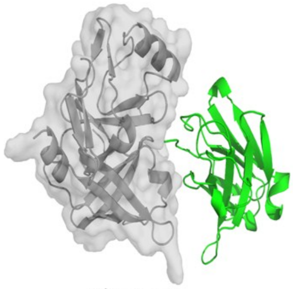

Cluster 1

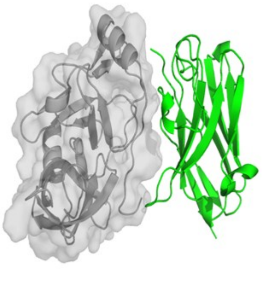

Cluster 8

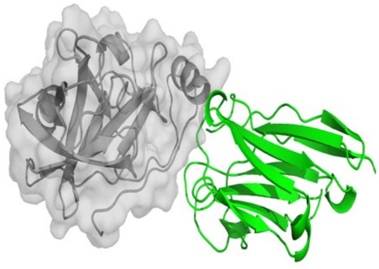

Cluster 6

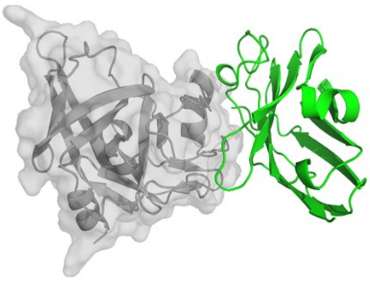

Cluster 11

Supplemental data 3. Modeled structure of NS3/4A showing catalytic triad, oxyanion loop and S1 pocket.

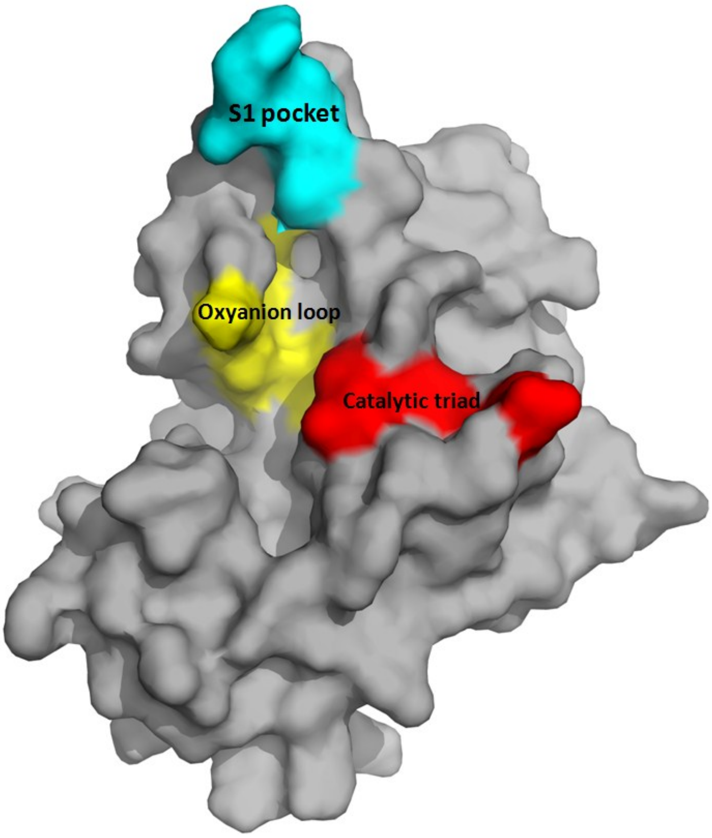

© 2015 by the authors; licensee MDPI, Basel, Switzerland. This article is an open access article distributed under the terms and conditions of the Creative Commons Attribution license (http://creativecommons.org/licenses/by/3.0/).
